# Evaluation of Embrace WetBond and Helioseal-F sealant retention with and without a Self-etch adhesive: A 12 month follow-up

**DOI:** 10.4317/jced.58707

**Published:** 2021-12-01

**Authors:** Gemimaa Mathew, Trophimus-Gnanabagyan Jayakaran, Hemalatha Ramkumar, Senthil Dakshinamoorthy, Shankar Paulindraraj, Nancy Solomon

**Affiliations:** 1 Consultant Pediatric Dentist. Rya Cosmo Hospital, Purasaiwakkam, Chennai – 600012, Tamil Nadu, India; 2 Senior Lecturer. Department of Pediatric and Preventive Dentistry, SRM Dental College, Ramapuram, Chennai – 600089, Tamil Nadu, India; 3 Professor and Head of the Department. Department of Pediatric and Preventive Dentistry, SRM Dental College, Ramapuram, Chennai – 600089, Tamil Nadu, India; 4 Reader. Department of Pediatric and Preventive Dentistry, SRM Dental College, Ramapuram, Chennai – 600089, Tamil Nadu, India

## Abstract

**Background:**

Pit and fissures on the young permanent tooth are ideal in harbouring dental plaque and calculus. Hence it is important to provide a preventive agent to protect against dental caries. Aim: To evaluate the retention of two different pit and fissure sealants with and without a self-etch adhesive in the first permanent molars for a period of one year.

**Material and Methods:**

280 molars were included among 70 healthy children. According to randomisation, the groups were divided into Group IA - Embrace without Adhse One F bonding agent and Group IB - Embrace with Adhse One F bonding agent, Group IIA - Helioseal F without Adhse One F bonding agent and Group IIB - Helioseal F with Adhse One F bonding agent. The sealants were assessed clinically at 3,6,9, and 12 months using the modified Colour, Coverage, Caries (CCC) sealant evaluation system.

**Results:**

At the end of 12 months, the retention rates of Group IB showed statistically significant results, followed by Group IIB, Group IA and Group IIA.

**Conclusions:**

At twelve months follow-up Embrase WetBond and Helioseal-F were better retentive when used with a Adhese One F bonding agent which was statistically significant.

** Key words:**Retention, Bonding agent, Embrace WetBond, Helioseal-F.

## Introduction

Young permanent molars have shown to be caries susceptible because of its complex morphology of pit and fissures (1). Food debris and bacteria from deep pits and fissures cannot be easily cleansed, as they have the highest caries susceptibility and this always remained a concern for the dentists (2). Hence the Sealing of these caries susceptible sites, which are inaccessible to routine oral hygiene practices, is considered as an effective method of preventing dental caries (3-6).

Various conservative ways of treating occlusal pits and fissures were studied in literature with zinc phosphate cement, (3) enamel fissure eradication, (4) prophylactic odontomy, (3) and ammoniacal silver nitrate, (7) but none had proved to achieve any great measure of success. Added to all the advantages, clinicians have faced a major challenge associated with effectively placing traditional Pit and Fissure Sealants as it is technique sensitive (8). High quality polymeric restorative materials are desirable especially when treating children with unpredictable tolerance, patience, and cooperation. (6)

In literature there are several studies which have reported an increased retention rate (8-11) and reduced effect of salivary microleakage (12) with the application of bonding agent. On the contrary there are a few studies which did not show an improved retention with the use of bonding agent before sealant application (9,13-16). A recently introduced seventh-generation bonding agent, Adhese Universal, performs disinfecting, priming, and bonding in a single step. The benefits of this procedure are that it increases patient comfort, reduces chair side time, decreases contamination, and increases efficacy, which would be promising in preventing pit-and-fissure caries in pediatric patients.

Owing to the sparse literature available regarding the effect of bonding agent (Adhese Universal, Vivapen, Ivoclar) and an introduction of new materials, makes continuing research on this subject even more necessary. Hence the aim of the present study was to evaluate the retention of two different pit and fissure sealants with and without a self-etch adhesive bonding agent in the first permanent molars for a period of one year.

## Material and Methods

-Ethical approval 

The research protocol was approved by the Institutional Review Board of the SRM Dental College, Ramapuram, Chennai with IRB number SRMDC/IRB/2014/MDS/No.801. The informed consent was received from the school principal and parents of the participating children.

-Inclusion criteria

• Age: 7-9 years 

• Frankl’s behaviour rating 3 and 4

• Newly erupted permanent molars 

• Within one year of post-eruptive period 

• Deep retentive pit and fissures

-Exclusion criteria

• Partially erupted teeth

• No evidence of incipient caries

• No evidence of inter-proximal caries 

• No evidence of occlusal caries

• No restorations in the teeth

• Molar incisal hypoplasia

• Fluorosis

• Developmental anomalies of the teeth

• Physically challenged children

-Study sample and technique.

The sample size was calculated based on previous studies (17) using G*Power 3.1 Version software with a power of 80% and confidence interval level 95%. A total of 100 children were screened for deep pits and fissures on all the first permanent molars under natural light with mouth mirror and explorer. A total of 280 first permanent molars were selected from 70 children based on the inclusion criteria, from whom parent consent was obtained. According to simple randomisation, the teeth were divided into 

Group I-A - Embrace WetBond (Pulpdent, USA.) without Adhse One F (Viva pen, Ivoclar Vivadent, India.) bonding agent

Group I-B - Embrace WetBond with Adhse One F bonding agent, 

Group II-A - Helioseal F (Ivoclar Vivadent, India.) without Adhse One F bonding agent 

Group II-B - Helioseal F with Adhse One F bonding agent.

The study was performed in the premises of a private school in Chennai. A portable dental unit (Chesa. Inc) which comprised of airotor, suction tip, 3-way syringe with a compressor unit aided with a portable dental chair (M.S. Surgicals) and LED light was used. All the children, underwent oral prophylaxis prior to the sealant placement. The tooth was isolated using cotton rolls and in Group I-A & Group II-A, the enamel surface was etched using 37% phosphoric acid (Prevest Denpro limited, India) for 15 sec, rinsed for 12-20 sec and dried. In Group I-A, Embrace Wetbond was applied to the lower right first permanent molar & in Group II-A - Helioseal F was applied to the lower left first permanent molar tooth surface according to the manufacturer’s instructions. After dispensing, the sealant on to the tooth surface, it is allowed to cover all pits and fissures and to extend onto the cusp ridges using an explorer. The final thickness upon application should be atleast 0.3mm. After application, the sealants were light cured for 20 seconds. In Group I-B & II-B, Adhse One F, which is a self-etch bonding agent available in the form of a pen is applied to the maxillary first molars and light cured for 20 sec. Following this in Group I-B, Embrace Wetbond was applied to the upper right first permanent molar & in Group II B - Helioseal F was applied to the upper left first permanent molar tooth surface as done Group I-A and II-A. All the samples were evaluated immediately for retention and seal of the occlusal surfaces. Visual/tactile examinations were performed in the dental chair using magnification loupes, mouth mirror and probe. The occlusion was checked using an articulating paper and high points if any were adjusted using a pear shaped composite finishing bur. All the children were instructed to refrain from eating and drinking for 30 min. A single operator performed all the sealant applications where 15 -18 children were treated in a day to avoid operator fatigue.

-Evaluation of the sealants.

The school children were re-examined and evaluated every 3rd, 6th, 9th and 12th month by a single investigator who was blinded on the method of sealant application, and had received training on evaluating the retention of Pit and fissure sealants and caries diagnosis using the modified Colour, Coverage, Caries (CCC) sealant evaluation system (18) ( Table 1).

**Table 1:**
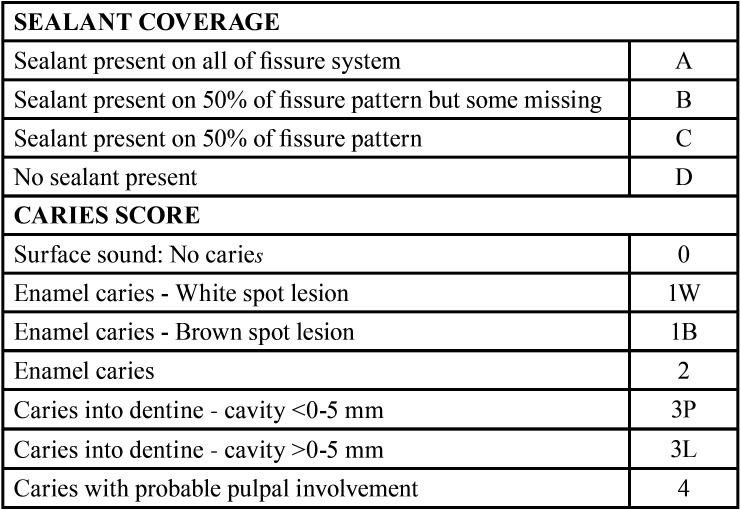
Summary of the Modified Colour, Coverage, Caries Sealant Evaluation System criteria.

-Statistical analysis.

Statistical analysis was performed using Statistical Package for Social Sciences (SPSS 20, IBM SPSS Statistics for Windows; version 20.0; IBM Corp., Armonk, NY, USA). Friedman’s test was used for intra-group comparison of sealant retention and caries scores at 3, 6, 9 and 12 months. The inter-group comparison of sealant retention and caries scores at 3, 6, 9 and 12 months was analysed using Wilcoxon signed‑rank test. The probability value < 0.05 was considered as statistically significant.

## Results

**Figure 1:**
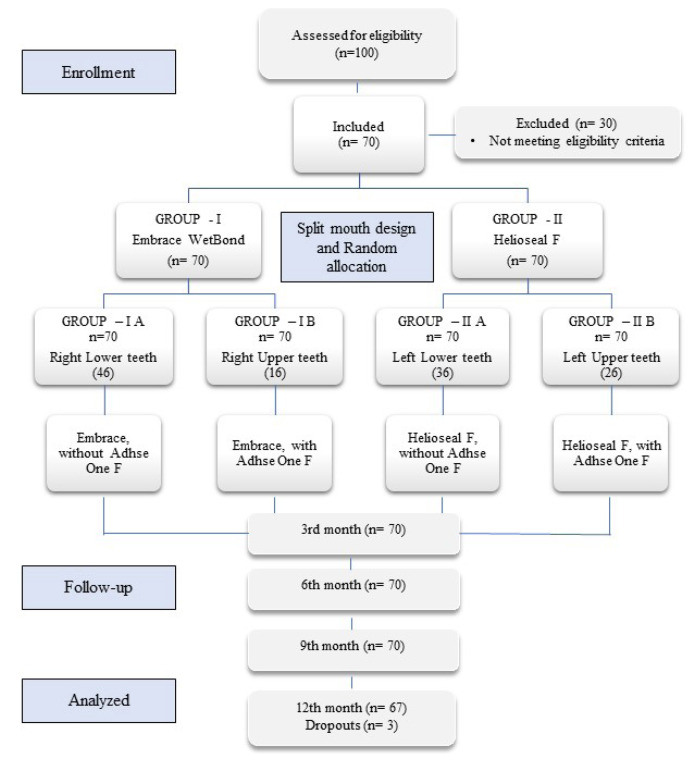
CONSORT flow diagram.

-Subjects and distribution:

At the beginning of the study, 70 children fulfilled the inclusion criteria and had received the sealants. The mean age of subjects examined at baseline was 8.39 ± 0.15 years. In all the four groups 70 teeth were examined each at 3, 6 and 9 months. At 12 months, follow-up was lost for 3 children because of the change in school, illness or absenteeism and so the number of teeth examined was 67 (Fig. 1). The sealants were assessed using the Modified CCC sealant evaluation system through visual clinical examination.

-Sealant Coverage:

Sealant coverage was deemed to be adequate if it was present on the entire fissure pattern (Code A). The retention of sealant in Group I-A (Embrace WetBond without Adhse One F bonding agent) during 3, 6, 9 and 12 months were 97%, 93%, 82% and 73.7%. For Group II-A (Helioseal F without Adhse One F bonding agent) the retention rates during 3, 6, 9, 12 were 97%, 84.6%, 75.3% and 64.7%. Group I-A (*P* =0.046) and Group II-A (*P* =0.037) showed a lower retention rate compared to Group I-B and II-B at the end of 12 months which was statistically significant ( Table 2).

**Table 2:**
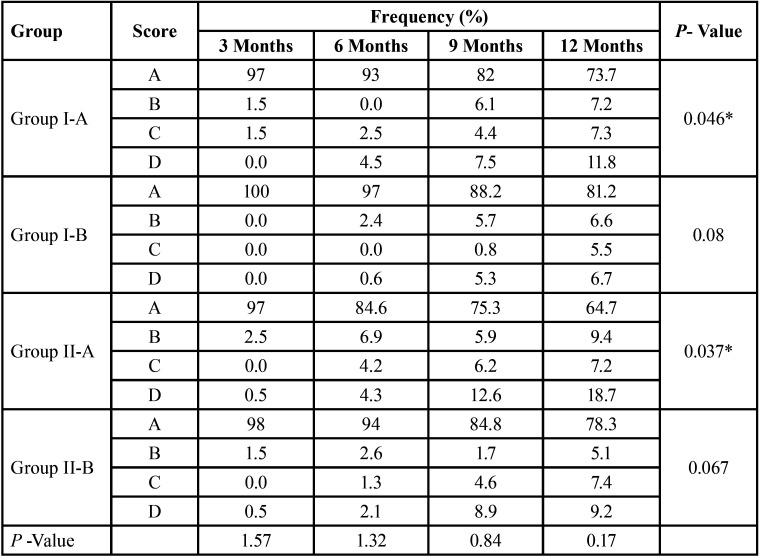
Comparison of Sealant Coverage of Embrace WetBond and Helioseal-F with and without bonding agent.

-Caries status of sealed surfaces:

The Caries free sound teeth (Score -0) at the end of 12 months in Group I-A, Group I-B, Group II-A and Group II-B were 82.7%, 86.4 %, 85.3% and 92.3%. The number of white-spot (score – 1W) and Brown spot (score -1B) lesions were more in Group I-A followed by Group II-A, Group I-B and Group II-B at the end of 12 months, but was not statistically significant ( Table 3).

**Table 3:**
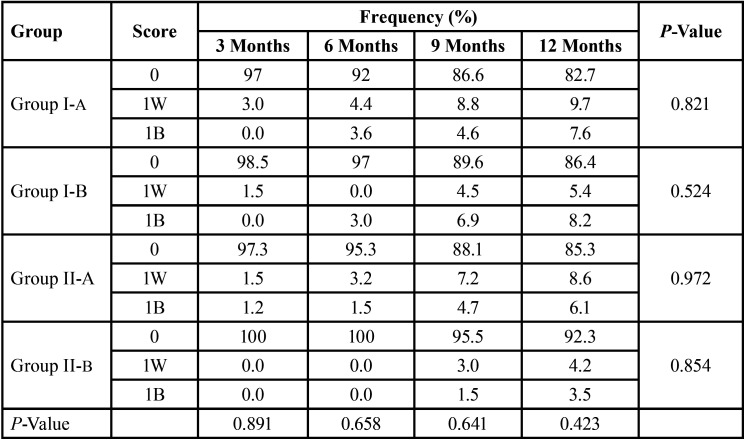
Comparison of Caries Scores of Embrace WetBond and Helioseal-F with and without bonding agent.

## Discussion

Several advancements in caries prevention have been studied over the past decades (19). The pit and fissure sealants have an increased acceptance by patients and is considered as an effective treatment in preventing dental caries (11,20-22).

The key aspect in the sealant application process is the isolation of teeth (8). In this study, both cotton roll isolation and evacuation tips were used. William and Mark recommended placing high volume evacuation tip against the secured rolls for a few seconds, instead of replacing it, will evacuate the excess moisture from the cotton (23,24). Isolating a tooth with rubber dam or cotton rolls are equally effective in retention rates of sealants (25). It has also been stated that absolute isolation is not necessary for the application of sealants as long as greater care is taken to avoid salivary contamination of the surface (24).

One of the most studied issues is whether a bonding agent should be placed before the sealant, in order to ensure its better retention on tooth enamel (26). The application of Bonding agent increases the retention of the sealants to pits and fissures by forming an intermediate layer between the etched enamel and the sealant (8). It also permits optimal infiltration (27) and formation of longer resin tags thus providing a micro‑mechanical retention to the sealant (28). Studies by Feigal *et al*. showed that the single‑bottle dentin bonding agents performed better (8,15). This is the first clinical trial using Adhese Universal, VivaPen, Ivoclar® which is a Self-etch single bottle bonding agent prior to pit and fissure sealant application. Adhese Universal possesses optimized mild-etching characteristics which effectively condition both un-etched and etched tooth surfaces; and due to its optimal balance of hydrophilic and hydrophobic monomers, it is highly tolerant towards dentin moisture thus permitting it to be suiTable for use with all etching protocols (29). The retention rates of the sealant at the end of this study, with and without bonding agent in both the groups clearly depicts that the application of bonding agent has enhanced the retention of the sealants. The results of the present study is however contrary to a study done by Zhang *et al*. in primary teeth, where the self-etching adhesive produced significantly lower sealant retention rates after 6, 12 and 18 months compared to phosphoric acid etching (30).

The present study followed a split-mouth design using Embrace WetBond and Helioseal F pit and fissure sealants where both sealant materials were to be applied in the same mouth to compare the material performance under similar environmental conditions (31). Embrace WetBond was found to be better retentive compared to Helioseal -F when used with Adhese universal bonding agent at the end of 12 months but was not statistically significant. Traditional sealants are hydrophobic, whereas Embrace WetBond is hydrophilic (32). Embrace Wetbond incorporates di-, tri- and multifunctional acrylate monomers into a sophisticated acid-integrating chemistry that’s activated by moisture. Once placed within the presence of moisture, the sealer spreads over the enamel surface. Exhibiting a distinctive chemistry, Embrace WetBond is miscible in water and flows into moisture-containing etched enamel and combines with it (33). A study done by Schlueter *et al*. showed contrary results, where 93% of Helioseal sealants and 27% of Embrace sealants were completely retained at the end of 12 months (34).

The prevalence of dental caries on sealed occlusal surfaces was zero at baseline. However, over a period of 12 months, the prevalence of white and brown spot lesions increased in Group I-A (17.3%) and Group II-A (14.7%), but was not statistically significant. Helioseal-F showed a decrease in caries incidence compared to Embrace WetBond. Analysis of the combined use of fluoride and dental sealants has shown retention of 92% after four years. This means that pit and fissure sealants confer additional caries preventive edges on the far side those of fluoride therapy alone (35). This might be the rationale why there was less caries prevalence when Helioseal F was used (Group II-A and II-B) at the end of the study.

The Limitations of the study include salivary contamination, though care was taken to restrict the saliva contamination by using cotton rolls and suction for each child, the ideal isolation technique of using rubber dam could not be followed since it was a large-scale study. Another limitation can be the follow‑up span of 1 year for the evaluation of caries development/ progression may be debated as a short span.

## Conclusions

At twelve months follow-up Embrace WetBond and Helioseal-F were better retentive when used with a bonding agent which was statistically significant. The prevalence of dental caries had reduced by the use of Helioseal-F when compared to Embrace WetBond, which was not statistically significant. Adhse One F undoubtedly proved to aid in the retention of the sealants which was clearly seen at the end of one year.
